# Disruption by SaCas9 Endonuclease of HERV-K*env*, a Retroviral Gene with Oncogenic and Neuropathogenic Potential, Inhibits Molecules Involved in Cancer and Amyotrophic Lateral Sclerosis

**DOI:** 10.3390/v10080412

**Published:** 2018-08-07

**Authors:** Gabriele Ibba, Claudia Piu, Elena Uleri, Caterina Serra, Antonina Dolei

**Affiliations:** Department of Biomedical Sciences, University of Sassari, Viale San Pietro 43B, 07100 Sassari, Italy; g.ibba@gmx.com (G.I.); claudia-piu@tiscali.it (C.P.); elenauleri@tiscali.it (E.U.); cserra@uniss.it (C.S.)

**Keywords:** human endogenous retroviruses, HERV-K*env*, CRISPR/Cas9 gene-editing, prostate cancer, amyotrophic lateral sclerosis, pathogenesis, SF2/ASF, TDP-43

## Abstract

The human endogenous retrovirus (HERV)-K, human mouse mammary tumor virus like-2 (HML-2) subgroup of HERVs is activated in several tumors and has been related to prostate cancer progression and motor neuron diseases. The cellular splicing factor 2/alternative splicing factor (SF2/ASF) is a positive regulator of gene expression, coded by a potent proto-oncogene, amplified, and abnormally expressed in tumors. TAR DNA-binding protein-43 (TDP-43) is a DNA/RNA-binding protein, negative regulator of alternative splicing, known for causing neurodegeneration, and with complex roles in oncogenesis. We used the clustered regularly interspaced short palindromic repeats (CRISPR)/Cas9 technology, with the Cas9 system from *Staphylococcus aureus* (SaCas9), to disrupt the HERV-K(HML-2)*env* gene, and evaluated the effects on cultured cells. The tool was tested on human prostate cancer LNCaP cells, whose HERV-K*env* transcription profile is known. It caused HERV-K(HML-2)*env* disruption (the first reported of a HERV gene), as evaluated by DNA sequencing, and inhibition of *env* transcripts and proteins. The HERV-K(HML-2)*env* disruption was found to interfere with important regulators of cell expression and proliferation, involved in manaling, RNA-binding, and alternative splicing, such as epidermal growth factor receptor (EGF-R), nuclear factor kappa-light-chain-enhancer of activated B cells (NF-κB), SF2/ASF, and TDP-43. These novel findings suggest that HERV-K is not an innocent bystander, they reinforce its links to oncogenesis and motor neuron diseases, and they open potential innovative therapeutic options.

## 1. Introduction

Human endogenous retroviruses (HERV) are around 8% of human DNA and are poorly and variably expressed by host cells. In general, they are highly defective, but a handful of complete proviruses has been described, with the classical genome organization of retroviruses [1,2]. Among cells of the same individual, the expression of different HERVs varies, depending on several factors. The first one is the availability to be transcribed of the chromatin region (DNA methylation, chromatin remodeling, etc.), where the HERV is located, which varies in relation to programs of development, cell histotype, cell differentiation and proliferation, oncogenesis, and/or tumor progression, among others. [3]. When the coding potential is retained, the retro-element undergoes the same regulation of surrounding cell genes. 

The HERV-K family gathered the attention of scientists because its HML-2 subgroup is the most recently integrated in the human genome, the least defective (91 full-length and near full-length proviruses), and the most active of the known HERVs [4,5], and is considered the most interesting HERV group to study in terms of potential oncogenic activity. Recently, increased expression of the HERV-K(HML-2) has been correlated with the progression of prostate cancer (PC) [6]. In addition, several PC cell lines natively express HERV-K*env* transcripts [7]. Of note, a study reported the anticancer role of a reverse transcription inhibitor (Abacavir), able to prevent the activity of HERVs reverse transcriptase [8]. Three mechanisms potentially involved in oncogenesis have been described: (i) The LTRs, which recruit transcription factors, and may enhance transcription of host genes. (ii) HERV-K-encoded potentially oncogenic proteins, such as Np9 (a critical molecular switch of multiple signaling pathways), and Rec (de-repressor of oncogenic transcription factors as the androgen receptor [9,10,11], both splicing product of the *env* gene. (iii) Recently, the cytoplasmic tail of HERV-K(HML2) Env, unique among the retroviral Env proteins tested, was found to have per se oncogenic properties, being a strong inducer of several transcription factors of the ERK1/2 pathway, and associated with cellular transformation [12].

The serine/arginine-rich (SR) proteins are positive splicing regulators and were shown to interfere with late RNA expression of the HIV retrovirus [13]. They are important in constitutive and alternative pre-mRNA splicing, and also modulate the expression of many oncogene and tumor-suppressor isoforms. One SR protein is SF2/ASF (splicing factor 2/alternative splicing factor), which targets approximately 1500 different mRNAs, including those involved in cell cycle regulation [14]. SF2/ASF is upregulated in various human tumors; *SFRS1* (*splicing factor*, *arginine*/*serine-rich 1*), the gene encoding SF2/ASF, is a potent proto-oncogene, with gene amplification and abnormal expression in many tumors. SF2/ASF, in turn, can inhibit onco-suppressive proteins [15,16,17,18,19,20,21,22]. Moreover, PC cells show higher levels of SF2/ASF, counteracted by the tumor suppressor miR-30c [23]. In LNCaP and other PC cells, SF2/ASF was identified as a disease-relevant effector of cyclin D1b, a cyclin deriving from alternative splicing of the CCND1 transcript, with markedly enhanced oncogenic functions not shared by full-length cyclin D1 [24]. In PC cells, SF2/ASF contributes to enhanced levels of constitutively activated androgen receptor splice variants, thus contributing to PC cell resistance to androgen-deprivation therapy [25].

We also focused on TDP-43 (TAR DNA-binding protein 43), a member of a large family of named RNA-binding proteins, which are important negative regulators of alternative splicing. It is involved in various aspects of cell proliferation and apoptosis, known for causing neurodegeneration [26] and with complex roles in oncogenesis, with both pro-oncogenic and anti-oncogenic effects in cells from different human tumors [27,28,29,30].

The CRISPR/Cas9 (clustered regularly interspaced short palindromic repeats/CRISPR associated protein 9) gene-editing technology nowadays is one of the most powerful tools to knockout an individual gene and to control its transcription, and rekindled the hopes in gene therapy. Therefore, as the expression of HERV-K Env was reported to be linked to important human diseases, like cancer, and recently also to neurodegeneration [31], we used the CRISPR/Cas9 gene-editing to disrupt the HERV-K(HML-2)*env* gene to evaluate the effects on cultured cells, with the ultimate aim to provide a tool for future innovative therapies.

## 2. Materials and Methods

### 2.1. Guide RNAs (gRNAs) Design

The *National Center for Biotechnology Information* (NCBI) website (https://www.ncbi.nlm.nih.gov/) was used for mining all the HERV-K(HML-2) family genome sequences, in search of conserved domains of the genome, in order to ensure the robustness of the tools to be designed. A full-length HERV-K genome is approximately 9.5 kb long [4], as are many retroviruses. In order to also catch sequences with large deletions, but still representative of a HERV-K(HML-2) genome, we collected all the sequences matching a query for HERV-K(HML-2) sequences of length >7000, and 49 reference sequences were obtained.

The conserved and variable portions of the viral genome were determined by a multiple sequence alignment in the Guidance V2 server (http://guidance.tau.ac.il/ver2/source.php), with standard parameters. The software rates each alignment, based on the robustness of an alignment to perturbations. The standard cut-off score of 0.93 was chosen, and all the residues below the cut-off value were replaced by an “N” in the reference sequence (Genebank accession ID: GU476574.2. Finally, the gRNAs analysis on the masked HERV-K(HML-2) genome was performed with the CRISPR/Cas9 designing tool on the Benchling website (https://benchling.com/). We set 20–22 nucleotides as the desired gRNA length; all the gRNAs targeted with PAMs (protospacer adjacent motifs) of the NNGRR form [32]. Sixty sequences were identified as possible targets for gRNAs. Among them, the top four with the highest “on target”–“off target” score, namely Km1, Km2, Km3, and Km4, were selected for the study.

### 2.2. Off-Target Analysis

To verify the specificity of our gRNAs, the top five predicted off-target regions in human genome were identified. Sets of primers were designed to amplify these regions; PCR amplification of human DNA was performed, and the amplicons were cloned into the pCR2.1 TA vector (Invitrogen). Ten clones for each off-target region were sequenced by the Sanger method (see next paragraph 2.3 for the method). No off-target effects were observed after analyzing the top five predicted off-target regions.

### 2.3. Cloning gRNAs

To create the SaCas9/anti-HERV-K*env*gRNA constructs, we used the existing pX601-AAV-CMV::NLS-SaCas9-NLS-3xHA-bGHpA;U6::Bsa1-sgRNA plasmid (Addgene, #61591), consisting of a *Staphylococcus aureus*-derived SaCas9/gRNA system, adapted for use in mammalian cells [33]. Protospacer regions corresponding to selected target sites were ordered, as a pair of 5′-G(N22)-3′ complementary oligonucleotides, containing Bsa1 overhangs at their respective 5′ ends. After annealing and phosphorylation by T4 polynucleotide kinase (New England Biolabs), double stranded protospacers were ligated into the Bsa1-digested pX601 backbone plasmid, which was dephosphorylated with calf intestine phosphatase (New England Biolabs, #M0290S). Bacterial clones were screened by PCR for the presence of gRNA protospacer inserts, using the top forward oligonucleotide of each annealed gRNA, in combination with reverse primer specific for the gRNA scaffold segment of U6-gRNA cassette. Successful clones were further verified by sequencing, using the same reverse primer.

### 2.4. Cells

The human prostate adenocarcinoma LNCaP cells (the American Type Culture Collection; #CRL-1740) were routinely maintained, as published [34].

### 2.5. DNA and RNA Extraction, Retrotranscription, and Real-Time (RT) PCR

Cellular DNA from 10^6^ cells was extracted by DNeasy Blood & Tissue Kit (Qiagen #69506). Aliquots of 300 ng of cell DNA were used for each DNA amplification, by standard methods [35]. The primer sequences used to amplify the region affected by the cut were as follows: fw: 5’-GGTAAGCGGGATGTCACTCAG-3′; rv: 5′-CAGAAGAGTGCAATGCAACTCC-3′. PolyA(+) RNAs were extracted from 50,000 cells, by mRNA Dynabeads kit (Dynal Biotech, #61011). Retrotranscription and selective amplification were performed as published [33], with the following primers: HERV-K*env* (fw: 5′-CTGAGGAATTGCAGGAGTT-3′, rv: 5′-GCTGTCTCTTCGGAGCTGTT-3′); epidermal growth factor receptor (EGF-R) (fw: 5′-ATGCCCGCATTAGCTCTTAG-3′, rv: 5′-GCAACTTCCCAAAATGTGCC-3′); nuclear factor kappa-light-chain-enhancer of activated B cells (NF-κB) p65 (fw: 5′-CTTGGCAACAGCACAGACC-3′, rv: 5′-GAGAAGTCCATGTCCGCAAT-3′); SF2/ASF (fw: 5′-GACATCGACCTCAAGAATCGC-3′, rv: 5′-GACCATACACCGCGTCTTCC-3′); TDP-43 (fw: 5′-TGGAGAAGTTCTTATGGTGCAGGTC-3′, rv: 5′-TCCATCTATCATATGTCGCTGTGAC-3′); small nuclear ribonucleoproteinD3 (SNRDP3) (fw: 5′-AAGTACTGCATGAGGCCGAG-3′, rv: 5′-CTTCAATGAGCTTCCCCCGA-3′). For each sample, the Ct (cycle threshold) value of the gene of interest was normalized by comparison to the Ct of the SNRDP3 housekeeping gene [36]. The data were reported as percentage of the relative mRNA expression, calculated on the normalized values according to the 2^−ΔCt^ method.

### 2.6. Transfection

The cells were plated in six-well plates at 90% confluency, and incubated overnight on Opti-MEM™ reduced serum medium (Gibco), to synchronize them at the G0 state. The next day, the cultures were co-transfected with a mixture of three plasmids: (i) the pLE-GFP-c1 plasmid (BD Bioscience Clontech), as transfection efficiency control; (ii) the pSpCas9(BB)-2A-Puro (PX459) V2.0 (Addgene plasmid # 62988) for the puromycin resistance [33]; and (iii) either the empty pX601-AAV-CMV::NLS-SaCas9-NLS-3xHA-bGHpA;U6::Bsa1-sgRNA (Addgene) or the SaCas9/anti-HERV-KenvgRNA construct, using the Lipofectamine^®^2000 reagent (Invitrogen), according to manufacturer’s protocol. After incubation for an additional 24 h, the transfection medium was replaced with fresh growth medium. The puromycin selection was performed by incubating the cells for one week in growth medium containing 2 µg/mL of puromycin (Sigma-Aldrich, Saint Louis, MO, USA). The medium was changed every three days for one week, to achieve maximum selection strength. Then, the selection medium was replaced with fresh growth medium; the cells were left to recover for one week, and then were harvested.

### 2.7. Western Blot Analysis

Cell extracts were prepared and processed as described [37]. The following antibodies were used to detect the proteins analyzed on this study: anti-human endogenous retrovirus type K envelope protein IgG monoclonal antibody (mAb, Austral Biological, #HERM-1811-5); anti-β-Actin mAb (Thermo Fisher Scientific, #BA3R); anti-Lamin A/C mAb (Thermo Fisher Scientific, #mab636); anti-human SF2/ASF mAb (Santa Cruz Biotechnology, #SC-33652); anti-human TDP-43 Ab (Proteintech, #10782-2-AP). All the primary antibodies were used at 1:1000, final concentration. Secondary antibodies were the following: goat anti-mouse IgG secondary antibody (Thermo Fisher Scientific, #31444); goat anti-rabbit IgG secondary antibody; horseradish peroxidase (HRP)-linked antibody (cell signaling technology, #7074), at 1:5000 final concentrations. After blottings, the membranes were developed with Supersignal West Femto Maximum sensitivity substrate (Thermo-Fisher Scientific Inc., #34095), and then exposed to Molecular Imager Versa Doc 3000 (BioRAD, Hercules, CA, USA). The stain intensity of the bands was acquired by QuantityOne Software (BioRAD, Hercules, CA, USA). Normalization of the target protein signal was performed on the β-actin or Lamin A/C housekeeper proteins, as required.

### 2.8. Statistics

Descriptive analyses included the computation of means, standard deviations (SD), and unpaired *t* test for independent samples.

## 3. Results

### 3.1. Setting and Validation of HERV-Kenv-Directed Guide RNAs

**Figure 1:**
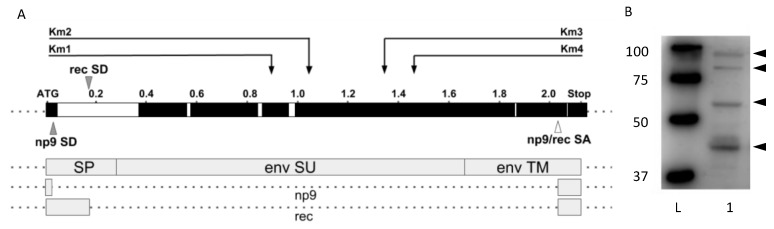
Schematic representation of the human endogenous retrovirus (HERV)-K*env* gene and of the spliced mRNAs, showing the targets for gRNAs, and the basal production of HERV-K Env proteins by the LNCaP cell line. (**A**) Map of the proviral HERV-K*env* gene, spanning from the ATG to the Stop codon. The singly spliced SU and TM *env* mRNA, the doubly spliced *np9* and *rec* mRNAs are shown as white boxes. The arrows indicate the targets of the Km1, Km2, Km3, and Km4 gRNAs. The arrowheads indicate the donor (grey arrowhead) and acceptor (white arrowhead) splicing sites, leading to the HERV-K*np9* and HERV-K*rec* mRNA splicing. (**B**) HERV-K Env proteins produced by LNCaP cells cultured for 48 h, as detected by Western blot assay. After blotting, the relevant bands were recognized by an anti-HERV-K Env mAb, as described in Methods. See Results for details. Lane 1: protein ladder (precision plus protein western C standards, Biorad, Hercules, California, USA), Lane 2: LNCaP cell lysate. Arrowheads: Env-specific bands. All the four HERV-K Env species are visible in this membrane.

To target the HERV-K*env* gene, we focused on the region spanning from 800 to 1500 bp from the HERV-K*env* gene ATG, because an in silico evaluation across the HERV-K genome sequences present in the GenBank^®^ NIH database showed that this region is well conserved. Moreover, the cut would not affect the other splicing *env* products, Np9 or Rec, as reported in Figure 1A. These sequences were screened for the presence of 20 to 22 nucleotides-long target regions, followed by NNGRR protospacer adjacent motifs (PAMs), which are specifically recognized by SaCas9 endonuclease [38]. Sixty possible targets were found in this region. Among them, four targets for gRNAs, with the highest “on target”–“off target“ score, were selected: the Km1 to Km4 gRNAs, located in the surface (SU) subunit of the predicted HERV-K Env protein, as indicated in Figure 1A, in which the coding domains of the *env* gene are also reported [39]. The gRNAs were cloned into a pX601-AAV-CMV::NLS-SaCas9-NLS-3xHA-bGHpA;U6::BsaI-sgRNA vector, which is an Adeno-associated virus (AAV) delivery vector, containing a 1 kb shorter orthologue of canonical *Streptococcus pyogenes* Cas9 (SpCas9), derived from *Staphylococcus aureus* (SaCas9). We decided to use an AAV vector, instead of the classical lentiviral vectors, because AAV has proven to be a safe and efficient vehicle to deliver therapeutic DNA to several tissue targets, and numerous studies have shown the potential of AAV-mediated delivery of genetic material into prostatic cancer cells [40].

To study the effect of HERV-K in the prostatic cancer and its putative links with the SF2/ASF protein, the LNCaP human cell line from a prostate adenocarcinoma was chosen, because transcripts from HERV-K loci occur commonly in prostate cancer cell lines, and both unspliced and spliced HERV-K*env* mRNAs have been described in these cells [7]. We tested the LNCaP cells for HERV-K*env* expression and confirmed the presence of HERV-K*env* mRNAs with primers designed by us. In addition, we were able to also show that these cells produce HERV-K Env proteins spontaneously, as detected by Western blot and staining with anti-HERV-K Env mAb; as shown in Figure 1B, the LNCaP cells produce spontaneously HERV-K Env proteins, with molecular weight (MW) of around 90, 80, 65, and 45 kDa, respectively. 

**Figure 2:**
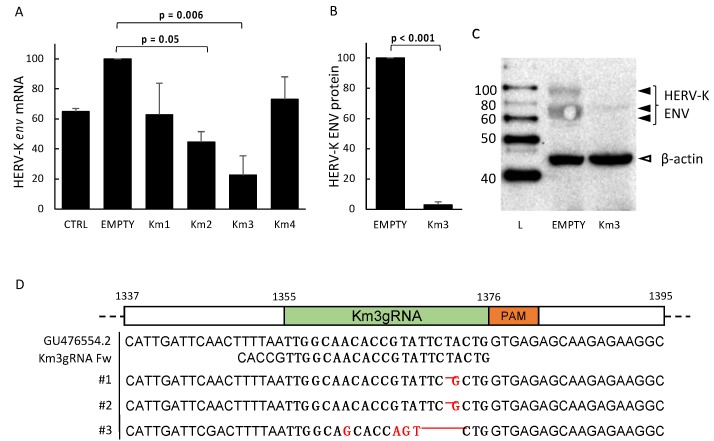
Expression of the HERV-K*env* gene in LNCaP cells exposed to the empty SaCas9 plasmid or to the pX601-Km(X) gRNA plasmids, and sequences of the targeted HERV-K*env* gene region in cells exposed to HERV-K*env* Km3 gRNA. (**A**) Mean HERV-K*env* mRNA levels in LNCaP cells exposed to the empty plasmid or to the pX601-Km(1-4) gRNA plasmids, as detected by real time (RT)-qPCR in five different experiments, run in duplicate, and reported as percent of the value of mock-transfected cells in each experiment. For controls of specificity, see Methods. (**B**) Mean levels of the HERV-K Env protein, as detected by Western blot in four different experiments performed with Km3 gRNA, run in duplicate; see Methods for details. Data are reported as mean intensity of the Env-specific stain of the band of interest, normalized with respect to that of the β-actin reference protein. (**C**) Row appearance of HERV-K Env bands in Western blot, as detected in a representative experiment with Km3 gRNA; (**D**) Sanger sequencing analysis on the genome region affected by the cut. CTRL: control; Empty: empty plasmid; Km1-4: Km(1-4) gRNAs. L: Ladder (MagicMark XP Western Protein Standard, Thermo Fisher Scientific); GU476574.2.: Reference sequence accession number; Km3 gRNA Fw: gRNA sequence including overhangs from the BsaI restriction enzyme; #1, #2, #3: Sequenced DNA coming from different bacterial colonies.

To test the ability of the designed gRNAs to induce site-specific cleavage in the HERV-K*env* gene, the LNCaP cells were transiently transfected with either the empty SaCas9 vector or with a SaCas9 plasmid carrying one of the four anti-HERV-K*env* gRNAs. The cultures were then selected by incubation in growth medium containing puromycin for one week. The selected cells were left to recover for an additional week in RPMI growth medium, and then were pooled, lysed, and processed for DNA, mRNA, and protein extraction, as reported in Methods. The polyA+ RNAs were retrotranscribed into cDNAs, which were amplified by real time PCR, with primers specific for the HERV-K*env* and the housekeeper SNRDP3 genes. Figure 2A reports the mean *env* mRNA levels in cells exposed to the gRNAs, as detected by RT-PCR. As shown, all the four gRNAs reduce the expression of HERV-K*env* mRNA with respect to the empty plasmid, with statistically significant reduction with the Km3 and Km2. Figure 2B reports the mean levels of the HERV-K Env protein in cells exposed to Km3 gRNA, as detected by Western blot, and evaluation of the stain intensity of the band of interest, normalized with respect to the reference β-actin protein. As expected, the efficiency of the gene-editing is higher at the protein level (mean 98% inhibition, *p* = 0.0004) than at the mRNA level (mean 76% inhibition, *p* = 0.006); this indicates that the damaged DNA can be transcribed in part, but that the transcripts are not suitable for the translation of Env proteins of the previously described size. In Figure 2C, the row appearance of HERV-K Env bands in a representative Western blot experiment with cells exposed to Km3 gRNA is reported.

To determine the nature of the reduced expression of the HERV-K*env* gene, DNA from aliquots of the same LNCap cells were amplified using primers covering the HERV-K*env* region interested by the cut. The amplicons were analyzed by Sanger sequencing, and the sequence obtained is reported in Figure 2D, indicating the indel mutation performed by Km3 gRNA.

### 3.2. Effects of HERV-Kenv Gene Disruption on Molecules Central to Signaling Networks

**Figure 3:**
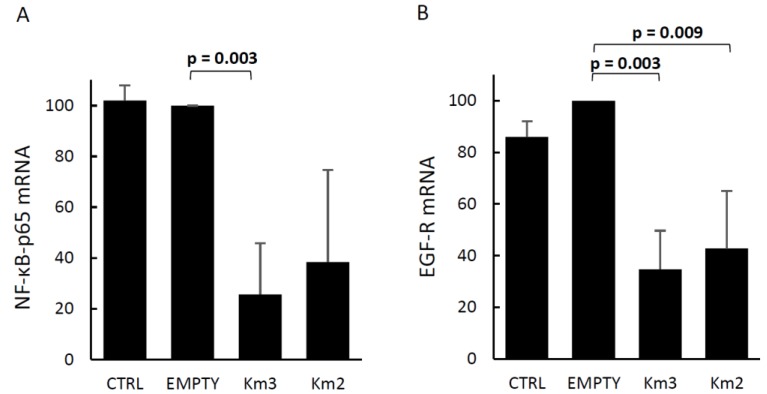
Levels of epidermal growth factor receptor (EGF-R) and nuclear factor kappa-light-chain-enhancer of activated B cells (NF-κB) p65 mRNAs in LNCaP cells transfected either with the empty plasmid or with the pX601-Km3 or Km2 gRNA plasmids. The same cDNAs and protein extracts used for the assays reported in Figure 2 were used. (**A**) Mean levels of EGF-R mRNA. (**B**) Mean levels of NF-κB p65 mRNA. Data are the means of the percent values with respect to those of empty-treated cells in each of three experiments, run in duplicate. For controls of specificity, see Methods.

To evaluate the possible effects of HERV-K*env* disruption on cell metabolism, the same cDNAs and protein extracts used for the assays reported in the previous paragraph were used to evaluate the expression of EGF-R (epidermal growth factor receptor) and of the p65 subunit of NF-κB (nuclear factor kappa-light-chain-enhancer of activated B cells). We selected these genes, because it was reported that HERV-K knockdown causes a major downregulation of EGF-R and NF-κB, which are molecules central to signaling networks pivotal for Ras-induced tumorigenesis, and block the expression of several tumor-associated genes [39]. As shown in Figure 3, the HERV-K*env* disruption by Km3 gRNAs is followed by the reduction of the levels of EGF-R mRNA (>60% inhibition, *p* = 0.003, Figure 3A) and of NF-κB p65 mRNA (80% inhibition *p* = 0.003, Figure 3B). Findings with the same trends were observed when the cells were exposed to the Km2 gRNA plasmid.

### 3.3. HERV-Kenv Gene Disruption Affects SF2/ASF and TDP-43 Proteins

Next, we focused on the effects of HERV-K*env* gene disruption on the expression of two important cellular regulators of gene expression, involved in signaling; RNA-binding; and various other aspects of transcription, including the alternative splicing: SF2/ASF (splicing factor 2/alternative splicing factor) and TDP-43 (TAR DNA-binding protein 43). The *splicing factor*, *arginine*/*serine-rich 1* (*SFRS1*), the gene encoding SF2/ASF, is a potent proto-oncogene, with abnormal expression in many tumors; SF2/ASF, in turn, can inhibit onco-suppressive proteins [16]. The TDP-43 protein is named after its capability to bind the TAR region of retroviruses; it is a RNA-binding protein, whose mutations, mislocalization, and aggregation are a pathological hallmark of ALS (amyotrophic lateral sclerosis) frontotemporal lobar degeneration [41]. It is involved in cell proliferation and has complex roles in oncogenesis [27,28,29,30]. Therefore, aliquots of the same cDNAs and protein extracts, utilized for the evaluations reported in the previous paragraph, were analyzed for the expression of SF2/ASF and TDP-43 mRNAs and proteins, respectively. 

**Figure 4:**
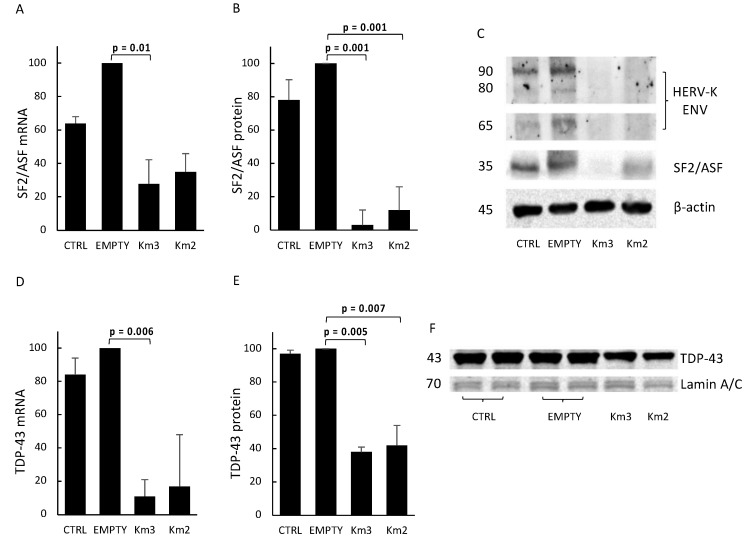
Levels of SF2 and TAR DNA-binding protein 43 (TDP-43) mRNAs and proteins in LNCaP transfected either with the empty plasmid or with the pX601-Km(3-2) gRNA plasmid. (**A**) Mean levels of splicing factor 2/alternative splicing factor (SF2/ASF) mRNA in three independent experiments, run in duplicate, reported as percent of the value of empty-treated cells in each experiment. For controls of specificity, see Methods. (**B**) Mean values of the SF2/ASF protein, as detected by Western blot in three different experiments. The data are reported as mean intensity of the specific stain of the band of interest, normalized with respect to that of the β-actin reference protein; see Methods for details. (**C**) Representative Western blot assay showing SF2/ASF and HERV-K Env proteins in LNCaP transfected either with the empty plasmid or with the pX601-Km(3-2) gRNA plasmid. After blotting, the relevant bands were recognized by anti-SF2/ASF and anti-HERV-K Env mAbs, respectively, as described in Methods. Lane 1: untreated LNCaP cells. Lane 2: LNCaP treated with empty SaCas9 plasmid. Lane 3: LNCaP treated with Km3 gRNA-SaCas9 plasmid. Lane 4: LNCaP treated with Km2 gRNA-SaCas9 plasmid. (**D**) Mean levels of TDP-43 mRNA, run in duplicate, and reported as percent of the value of empty plasmid-treated cells in each experiment. For controls of specificity, see Methods. (**E**) Mean values of the TDP-43 protein, as detected by Western blot in three different experiments. Evaluated as mean intensity of the stain of the band of interest, normalized with respect to that of the reference protein; see Methods for details. In (**E**,**F**), to ensure equal protein loading, the anti-Lamin A/C mAb was used, instead of the anti-β-Actin mAb, to avoid the overlapping of the staining of two proteins of similar MW. (**F**) Representative Western blot assay showing TDP-43 protein, as recognized by an anti-TDP-43 pAb, as described in Methods. Lanes 1–2: untreated LNCaP cells. Lanes 3–4: LNCaP treated with gRNA-free SaCas9 plasmid. Lane 5: LNCaP treated with Km3 gRNA-SaCas9 plasmid. Lane 6: LNCaP treated with Km2 gRNA-SaCas9 plasmid.

The data on SF2/ASF expression are reported in Figure 4A–C. The results of real time PCR amplification of cDNAs with primers specific for SF2/ASF are reported in Figure 4A; as shown, HERV-K*env* disruption caused up to 72.2% reduction of SF2/ASF mRNA levels (Km3 gRNA, *p* = 0.01). Figure 4B reports the normalized data of the mean levels of the SF2/ASF protein, as detected in three different Western blotting experiments, and indicates an average >95% reduction of the normalized stain intensity of the SF2/ASF protein after exposure to both Km(3–2) gRNAs (*p* = 0.001). Figure 4C shows a representative Western blot experiment, with the levels of SF2/ASF protein in cells exposed or not to the HERV-K*env* gene-editing. As shown, cell exposure to the Km3 gRNA or to Km2 gRNA plasmids caused an almost complete disappearance of the SF2/ASF protein, while the housekeeping β-actin protein band was similar to that of the cells exposed to the empty plasmid.

The data on TDP-43 expression are reported in Figure 4D–F. As shown in Figure 4D, Km3 gRNA-mediated HERV-K*env* disruption caused an average 90% reduction of TDP-43 mRNA levels, compared with that of the cells exposed to the empty plasmid (*p* = 0.006). In Figure 4E are reported the mean levels of the TDP-43 protein, normalized with respect to the Lamin A/C reference protein, as detected in three separate experiments, indicating an average halving of the normalized stain intensity of the TDP-43 protein (*p* = 0.005). To avoid overlapping of the TDP-43 band (MW 43,000) with that of the β-actin reference protein (MW 45,000), the Western blot membrane was stained for the Lamin A/C protein (MW 69,000–70,000). Figure 4E reports the levels of the TDP-43 protein in cells exposed either to the empty plasmid or to the Km(3-2) gRNA plasmid, in a representative Western blot experiment. As shown, after HERV-K*env* gene-editing, a significant reduction also of the TDP-43 protein occurred, while the housekeeping protein band was similar to that of the cells exposed to the empty plasmid or of the untreated control cells.

When the cultures were exposed to the Km3 gRNA plasmid for 24 h only, instead of 48 h, the reduction of HERV-K*env* was already detectable, while the levels of SF2/ASF and TDP-43 were unchanged (not shown). This observation is in keeping with the possibility that the effect on SF2/ASF and TDP-43 is consequential to HERV-K*env* disruption, and not an off-target result. The possibility that the effects on the expression of EGF-R, NF-κB, SF2/ASF, and TDP-43 could be due to off-targets was excluded also because they were obtained at both the mRNA and protein levels by gene-editing with the Km2 gRNA, as a consequence of disruption of the HERV-K*env* gene.

## 4. Discussion

The CRISPR/Cas9 technology is undergoing unprecedented expansion as a top genome editing technology. This procedure allows an easy knockout of an individual gene, to control its transcription and effects, and rekindled the hopes in gene therapy for new treatments for a number of diseases of genetic, infectious, or neoplastic origin [42,43,44], provided that patient’s individual genomes are considered, to minimize the risk of treatment failure and/or adverse outcomes [45]. A relevant achievement, so far, was the successful eradication of the HIV-1 retrovirus, both in vitro and in animal models [46,47]. The first trial by CRISPR gene-editing on humans occurred in China for lung carcinoma, and many other trials are planned worldwide [48].

The present paper reports the first disruption by gene-editing of a gene of a human endogenous retrovirus (HERV-K); the validation of the tool; and some novel effects, which may be relevant for the pathogenic mechanisms of human diseases that have been connected with this retro-element, especially prostate cancer and ALS.

We focused on HERV-K because, of the dozens of HERV families, only HERV-W and HERV-K were repeatedly reported to have potentially pathogenic properties, both linked to the proteins coded by their *env* genes. The HERV-W is linked to multiple sclerosis [49,50], and the HERV-K to cancer [5,51] and amyotrophic lateral sclerosis [52,53,54]. 

To set the tool against HERV-K*env*, we decided to target a conserved domain of the *env* gene, in order to potentially catch all the HERV-K*env* sequences considered for the study, and downstream the domain, which generates, by *env* splicing, the Rec and Np9 transcripts/proteins (Figure 1A). In fact, these spliced HERV-K*env* transcripts are expressed in a variety of different normal human tissues, and a functional relevance in normal human cell physiology was suggested [55]. Of the top four gRNAs selected for best “on target”–“off target” score from a total of sixty possible targets, the Km3 gRNA was selected, because it was the most efficient in affecting HERV-K*env* at the DNA, mRNA, and protein levels.

The LNCaP cells were chosen as a model of prostatic cancer cell, because their expression of HERV-K*env* transcripts was described previously [7]. We confirmed that these cells produce HERV-K*env* mRNAs. In addition, here we show for the first time that in LNCaP cells, the HERV-K*env* gene spontaneously undergoes a complete expression, up to the production of four proteins recognized by the anti-HERV-K Env mAb in Western blot (Figure 1B), of MW around 90, 80, 65, and 45 kDa, respectively. It seems likely that the 90 kDa protein is the full-length unprocessed precursor with its signal peptide; the 80 kDa could be the glycosylated full-length precursor or the unglycosylated full-length precursor with the signal peptide; the 65 kDa is the glycosylated SU subunit, and the 45 kDa the unglycosylated SU subunit. These MW values are in line with the values described in human 293T embryo kidney cells, HERV-K*env*-transfected to produce simian immunodeficiency virus/HERV-Kenv pseudotypes [56].

The LNCaP cells showed that the Km3 gRNA plasmid behaved as a valid tool for the disruption of the HERV-K*env* gene. In fact, when DNA from cells were exposed to the Km2 or to the Km3 gRNA plasmids, the *env* gene was affected, with almost complete disappearance of the HERV-K Env protein in Western blot, indicating that even though the damaged HERV-K*env* DNA can be transcribed in part, the transcripts are not suitable for the translation of Env proteins of the previously described size (Figure 1B–D).

The link between HERV-K and cancer has been proposed for decades [57]. Presently, increased HERV-K expression in tumors is associated with shorter relapse-free survival [58]. The HERV-K Env protein was shown to promote key signaling pathways, involved in cellular movements, cancer, cell death/survival, and cell proliferation, with a central role in regulating the RAS-ERK-RSK signaling pathway [39,52]. After HERV-K knockdown, breast cancer cells undergo a reversion to a non-tumorigenic phenotype, with a major downregulation of EGF-R and NF-κB [39]. Overexpression of EGF-R and its downstream pathway are linked to epithelial–mesenchymal transition, migration, and tumor invasion [59], while NF-κB plays a key role in regulating the immune responses and promotes cancer-initiating cells [60]. Therefore, the evaluation of these two proteins in HERV-K*env*-disrupted cells seemed to us a good assay for the validation of the CRISPR/Cas9 HERV-K*env* gene-editing tool. The observed downregulation of EGF-R and NF-κB (Figure 3) confirms the link between the expression of HERV-K*env* and both EGF-R and NF-κB, thus validating the tool, and reinforces the links between the HERV-K retroelement and pivotal signaling pathways, central to cancer and immune responses.

Of relevance is the novel finding, reported in Figure 4A–C, that HERV-K*env* disruption dowregulates the expression of SF2/ASF, a member of the serine/arginine-rich protein family, which definitely plays important roles in inflammatory disorders and cancer, through signaling; RNA-binding; and various other aspects of transcription, including alternative splicing. SF2/ASF is considered a potent proto-oncogene, abnormally expressed in many tumors, and which can inhibit oncosuppressive proteins [16,19], but is also able to regulate neuropathogenic viruses, such as JCV and HIV [61,62]. Therefore, our finding of a connection between the expression of HERV-K*env* and that of SF2/ASF reinforces the possibility that the expression of HERV-K*env* in many tumors [6,51,63,64,65,66] and in HIV-positive persons [67,68] is more that the mere expression of an innocent bystander during diseases such as cancer and HIV infection.

Of note is also the novel link between HERV-K Env and TDP-43 reported in this paper. The latter protein is considered a hallmark of ALS. The multiple pathogenic mechanisms of ALS (including TDP-43 mutations) share cytoplasmic TDP-43 deposits as common trait, which are thought to be critical in the degenerative process of motor neurons. A dysregulated TDP-43 production was proposed to be the cause of TDP-43 accumulation in the cytoplasm and loss of transport to the nucleus [69,70]. The TDP-43 protein is involved in the control of cell proliferation, and has complex roles in oncogenesis. Both pro-oncogenic and anti-oncogenic effects of TDP-43, or both, were reported in cells from different human tumors. TDP-43 overexpression promoted tumor progression and autophagy in glioblastoma [71]; its silencing impaired the migration and invasion of non-small cell lung cancer cells in vitro [72]; in melanoma, TDP-43 was proposed as a novel oncogene able to regulate the cell proliferation and metastasis [27]. On the other hand, a TDP-43 fragment was pro-apoptotic in breast cancer cells [30], and TDP-43 overexpression was related to good prognosis of neuroblastoma and breast cancer patients [28]. Moreover, depending on the specific miRNA affected, TDP-43 may play multifaceted roles in migration and survival of lung cancer cells. By regulating miR-423-3p, TDP-43 may promote migration of lung cancer cell; in contrast, TDP-43 increases miR-500a-3p expression, whose reduction is associated with poor survival of lung cancer patients, suggesting that TDP-43 may have a suppressive role in cancer [30].

Here, we provide novel evidence that the disruption of the HERV-K*env* gene causes a statistically significant reduction of TDP-43 expression, at both the mRNA and protein level, as shown by Figure 4D,E. This novel finding reinforces the relationship between HERV-K and TDP-43, which is involved in mRNA stability, RNA splicing, microRNA processing, transport, and local translation.

An average halving of TDP-43 levels by HERV-K*env* disruption is a relevant finding, because cellular functions of TDP-43 are critical for cell survival, and optimal levels of the TDP-43 protein are maintained by an autoregulatory pathway [73]. Moreover, levels of TDP-43 (either wild-type or mutant protein) increased by less than a factor of 2 are highly deleterious, and lead to neurodegeneration [74,75].

Our novel findings provide evidence of an intriguing scenario, in which HERV-K is not only a target of TDP-43 regulation, but is able also to interfere with this important regulator of cell expression. Because HERV-Kenv-transgenic mice die of an ALS-like disease [31], our data reinforce the links not only between HERV-K and oncogenesis, but also between HERV-K and ALS. If there is a contribution of HERV-K*env* expression to ALS pathogenesis, this would open potential therapeutic options for this devastating no-therapy disease.
